# Antitumor Activity of Hierridin B, a Cyanobacterial Secondary Metabolite Found in both Filamentous and Unicellular Marine Strains

**DOI:** 10.1371/journal.pone.0069562

**Published:** 2013-07-29

**Authors:** Pedro N. Leão, Margarida Costa, Vitor Ramos, Alban R. Pereira, Virgínia C. Fernandes, Valentina F. Domingues, William H. Gerwick, Vitor M. Vasconcelos, Rosário Martins

**Affiliations:** 1 CIIMAR/CIMAR – Interdisciplinary Centre of Marine and Environmental Research, University of Porto, Porto, Portugal; 2 Scripps Institution of Oceanography, University of California, San Diego, La Jolla, California, United States of America; 3 REQUIMTE, Instituto Superior de Engenharia, Instituto Politécnico do Porto, Porto, Portugal; 4 Skaggs School of Pharmacy and Pharmaceutical Sciences, University of California, San Diego, La Jolla, California, United States of America; 5 Department of Biology, Faculty of Sciences, University of Porto, Porto, Portugal; 6 Centre of Health and Environmental Research – CISA, Superior School of Health Technology of Porto, Polytechnic Institute of Porto, Vila Nova de Gaia, Portugal; 7 Institute for Molecular and Cell Biology – IBMC, University of Porto, Porto, Portugal; University of New South Wales, Australia

## Abstract

Cyanobacteria are widely recognized as a valuable source of bioactive metabolites. The majority of such compounds have been isolated from so-called complex cyanobacteria, such as filamentous or colonial forms, which usually display a larger number of biosynthetic gene clusters in their genomes, when compared to free-living unicellular forms. Nevertheless, picocyanobacteria are also known to have potential to produce bioactive natural products. Here, we report the isolation of hierridin B from the marine picocyanobacterium *Cyanobium* sp. LEGE 06113. This compound had previously been isolated from the filamentous epiphytic cyanobacterium *Phormidium ectocarpi* SAG 60.90, and had been shown to possess antiplasmodial activity. A phylogenetic analysis of the 16S rRNA gene from both strains confirmed that these cyanobacteria derive from different evolutionary lineages. We further investigated the biological activity of hierridin B, and tested its cytotoxicity towards a panel of human cancer cell lines; it showed selective cytotoxicity towards HT-29 colon adenocarcinoma cells.

## Introduction

Marine cyanobacteria have been shown to produce a diverse array of biologically significant natural products with activity in models for anticancer, neuromodulatory and anti-inflammatory drug discovery, and other areas [1]. Benthic, filamentous forms, in particular members of the classical (botanical) orders Oscillatoriales and Nostocales, have been the major sources of secondary metabolites reported from marine cyanobacteria [2]. This is in part explained by the fact that filamentous and colonial cyanobacteria appear to have larger genomes and thus can better accommodate sizable polyketide and non-ribosomal peptide pathways than picocyanobacteria [3], [4]. These classes of biosynthetic products make up the majority of secondary metabolites isolated from cyanobacteria thus far [2], although a previously unrecognized capacity to produce ribosomally-encoded modified peptides has recently been described [5], [6]. Another consideration is that some filamentous and colonial cyanobacteria grow to relatively high densities in coastal ecosystems, such as in mats or macroscopic tufts, thus yielding enough biomass for chemical investigations from environmental samples.

Conversely, unicellular cyanobacteria usually need to be cultured in order to produce sufficient biomass for chemical and biological studies. The lack of “chemistry-ready” environmental samples and the difficulty to bring certain strains into laboratory culture may have skewed our perception of the richness of such smaller genome size cyanobacteria in terms of secondary metabolite production. As an example, the marine picocyanobacterium *Prochlorococcus*, which is widespread in open ocean waters, produces a complex set of polycyclic lantipeptides [6]. The unicellular, not-yet-cultured symbiotic cyanobacterium *Prochloron didemni* is also known to produce a diverse array of secondary metabolites, including cyclic peptides and unusual fatty acids [7]. It should be noted that both of these examples report metabolites of a ribosomal origin, which may be a trend in picocyanobacteria as these more compact biosynthetic gene clusters may be better accommodated in small genomes.

The bioactive potential of picoplanktonic marine cyanobacteria has also been investigated by our group, with a focus on several *Synechococcus* and *Synechocystis* strains isolated from the Portuguese coast [8]. A recent survey of cyanobacterial genomes [4] indicated the presence of biosynthetic gene clusters, mainly of the polyketide synthetase (PKS) and bacteriocin variety, among picocyanobacteria genera *Cyanobium*, *Synechococcus* and *Prochlorococcus*.

**Figure 1:**
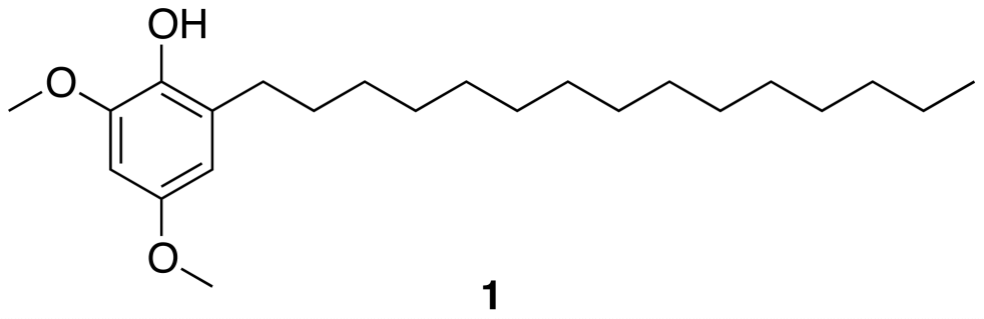
Structure of hierridin B (1).

Here, we report the NMR-based isolation of hierridin B (**1**, Fig. 1) from the picocyanobacterium *Cyanobium* sp. LEGE 06113 which had been isolated from the Atlantic coast of Portugal. The structure of **1** was confirmed by NMR and MS analyses. To our knowledge, this is the first report of a secondary metabolite from this cyanobacterial genus. Compound **1** had previously been isolated from the marine filamentous cyanobacterium *Phormidium ectocarpi* strain SAG 60.90 and showed antiplasmodial activity [9]. We show that both cyanobacterial strains possess polyketide synthase (PKS) biosynthetic machinery, which is predicted to be involved in the biosynthesis of hierridin B. To further investigate the biological properties of this metabolite, we treated a panel of eight human cell lines to this compound. Interestingly, when tested up to a maximum concentration of 30 µg mL^−1^ (82.3 µM), hierridin B was only active against the colorectal adenocarcinoma cell line HT-29.

## Results

### Isolation and identification of hierridin B

**Figure 2:**
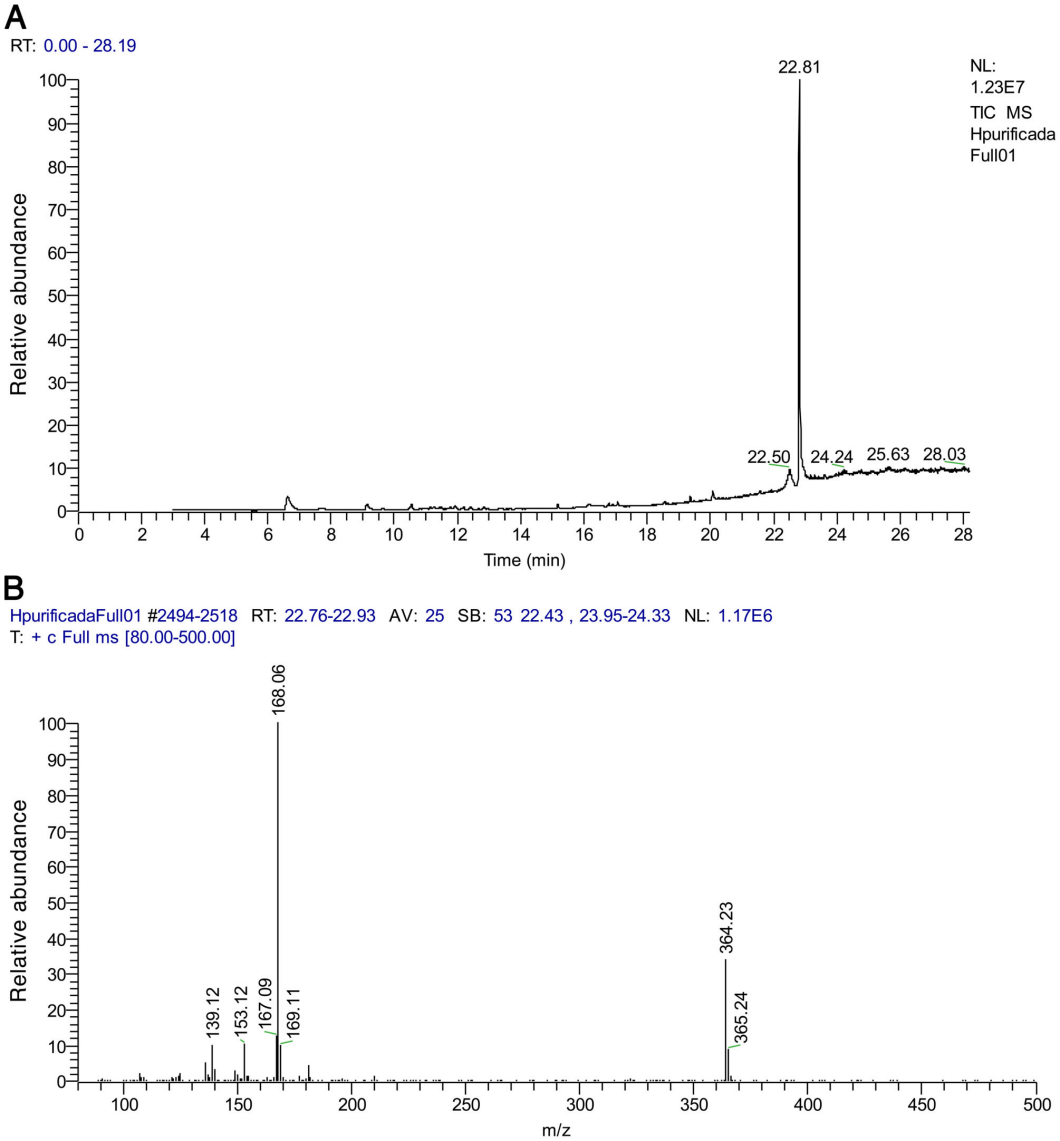
GC-MS analysis of purified 1. Top panel – total ion chromatogram (TIC). Bottom panel – mass spectrum of the main chromatographic peak (RT = 28.1 min), with the diagnostic ions for hierridin B at *m/z* 364 and 168 clearly seen.

A crude lipophilic extract from *Cyanobium* sp. LEGE 06113 was fractionated using vacuum-liquid chromatography (VLC). The ^1^H NMR spectrum (500 MHz, CDCl_3_) of one of the most non-polar fractions contained two sharp singlets at δ3.85 and δ3.76, suggestive of aromatic methoxy groups, which led us to further investigate this fraction and ultimately obtain compound **1** after purification by reversed-phase (RP) HPLC. Identification of the compound was initially based on comparison of the ^1^H NMR data with literature values for hierridin B and for one extended chain analogue, which had been previously reported [9] (Fig. S1). The length of the aliphatic chain of the isolated compound could not, however, be rigorously derived from the integration of the ^1^H NMR signals corresponding to the methylene envelope at δ1.30-1.23. Consequently, GC-MS data of the compound were acquired (no ionization was observed under our standard LC-ESI-MS conditions) and the identity of the purified metabolite was confirmed as hierridin B, due to the characteristic ions observed at *m/z* 364 [C_23_H_40_O_3_], *m/z* 168 [C_9_H_12_O_3_] (aromatic moiety following benzylic fragmentation and McLafferty rearrangement) and m/z 167 (benzylic moiety) (Fig. 2). Papendorf et al. [9] had used mass spectrometry data to aid in the structural characterization of metabolite **1** following its partial purification from *P. ectocarpi* SAG 60.90. In the previous work with *P. ectocarpi*, the hierridins were obtained as a 1∶1 mixture of **1** (pentadecyl-containing) and its heptadecyl analogue, both of which yielded the same fragment mass at *m/z* 168, but for which a molecular ion was observed at *m/z* 392 for the larger compound. We saw no evidence for the heptadecyl-containing metabolite in our materials from *Cyanobium* sp. LEGE 06113, nor could we find evidence for the presence of any other structurally related compound.

### Morphological and phylogenetic analyses

**Figure 3:**
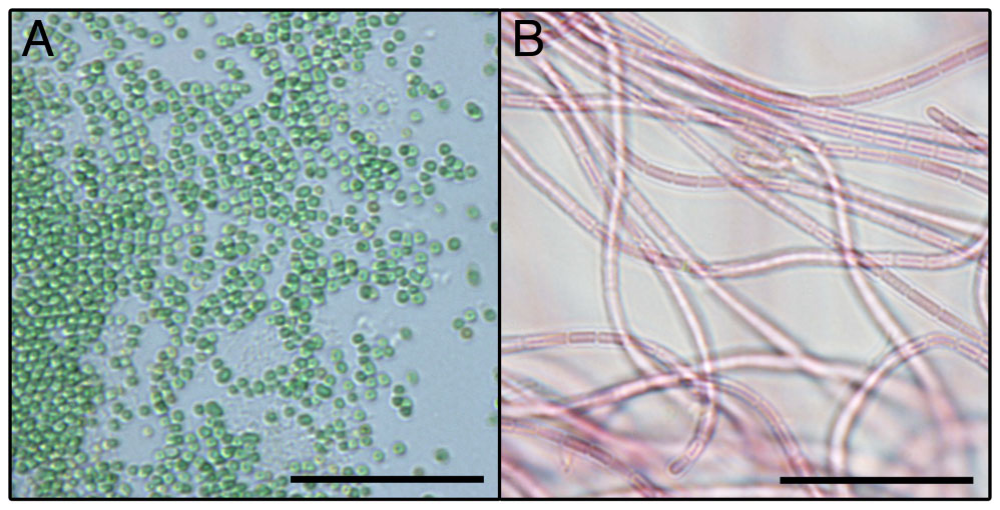
Microphotographs of the two cyanobacterial strains known to produce 1. A – *Cyanobium* sp. LEGE 06133; B – *Phormidium ectocarpi* SAG 60.90. Scale bar = 20 µm.

**Figure 4:**
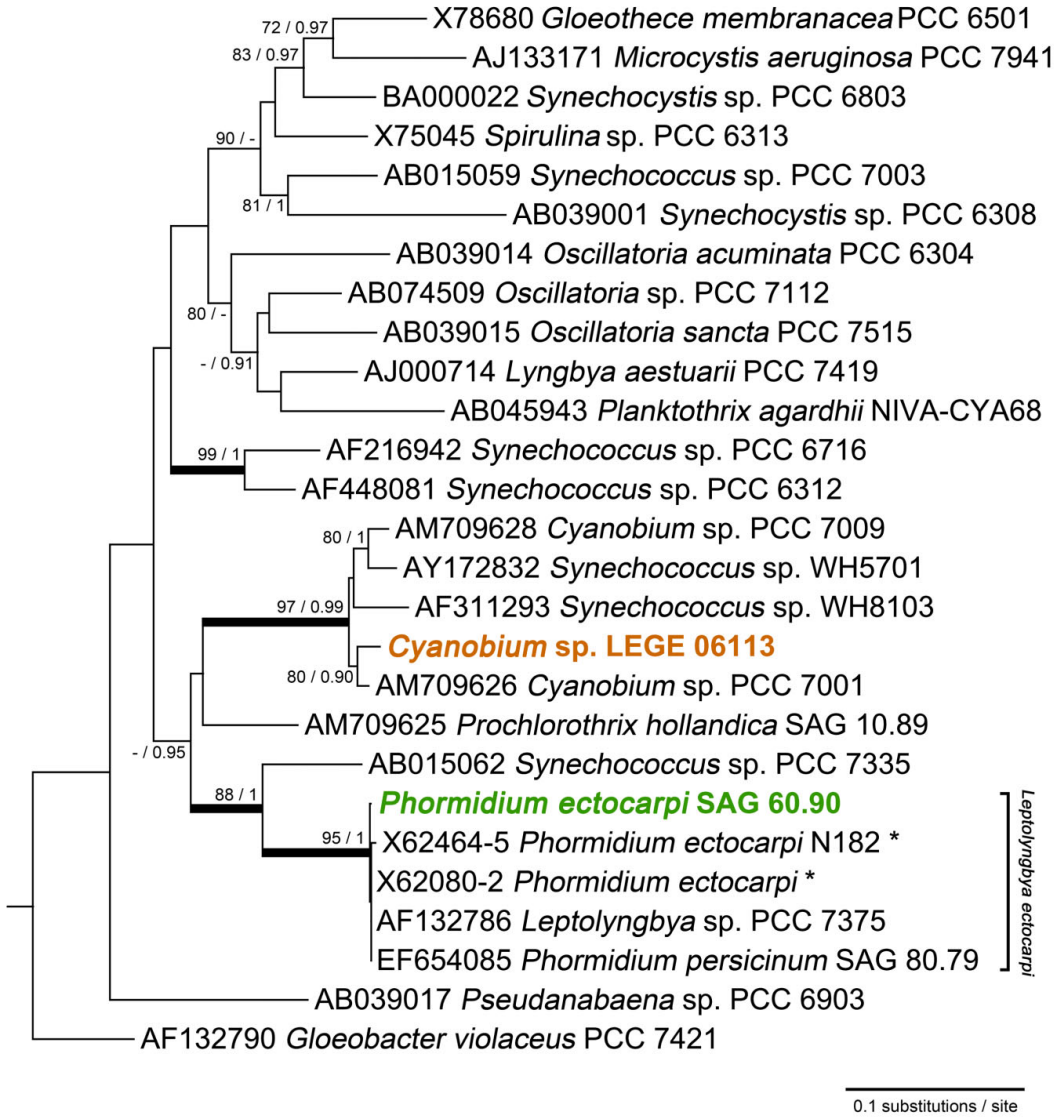
Maximum likelihood tree of 16S rRNA gene sequences from selected cyanobacteria and from the two strains known to produce metabolite 1. The nodal support values indicated near internal branches were determined by ML and BI methods, respectively; bootstrap values (for ML) below 70% and posterior probability values (for BI) below 0.90 were omitted. Thick branch lines indicate clades (or sub-clades) that are supported by simultaneous ≥85% bootstrap values and ≥0.95 bayesian posterior probabilities. The tree was rooted using *Chloroflexus aurantiacus* J-10-fl (D38365) as the outgroup, which was removed for clarity. Asterisks indicate concatenated sequences.

Cells from *Cyanobium* sp. LEGE 06113 (Fig. 3A) are pale blue-green, spherical to shortly rod-shaped, with a diameter of 0.9±0.1 µm (n = 20). Based on these morphological characteristics, the cyanobacterium was identified as *Cyanobium* sp., according to Komarék and Anagnostidis [10]. This identification is also in accordance with the criteria of the Bergey's Manual of Systematic Bacteriology [11]. Since hierridin B had been previously reported from the filamentous cyanobacterial strain *P. ectocarpi* SAG 60.90 (see morphology comparison in Fig. 3), we performed a 16S rRNA gene phylogenetic analysis to clarify the evolutionary relatedness between the two hierridin B producing cyanobacterial strains. From the phylogenetic tree (Fig. 4), it is clear that *Cyanobium* sp. LEGE 06113 is located in the same clade as other picocyanobacteria. This strain forms a sub-clade with the reference strain *Cyanobium* sp. PCC 7001; interestingly, the PCC 7001 sample was also isolated primarily from intertidal sediments [11]. This analysis also identifies that *Phormidium ectocarpi* SAG 60.90 is very closely related to other marine, phycoerythrin-rich filamentous cyanobacteria, including the reference strain *Leptolyngbya* sp. PCC 7375 (cluster 4, [11], [12]; formerly assigned to *P. ectocarpi* [13]). However, it is also clear that all of the above strains are encompassed within a wider clade that also includes the picocyanobacterium *Synechococcus* sp. PCC 7335. It should be noted that the clustering of the two clades that comprise each of the two hierridin B producers has no phylogenetic strength, because it lacks statistical support (ML bootstrap value of 44%) (Fig. 4).

### Presence of PKS genes

We have employed a PCR-based approach to detect the presence of PKS-related genes, by using degenerate primers targeting ketosynthase (KS) domains [14]. This allowed for the detection of one type I PKS gene fragment in *Cyanobium* sp. LEGE 06113 and two type I PKS gene fragments in *P. ectocarpi* SAG 60.90. The amino acid sequence identity between the two *P. ectocarpi* PKS genes was 54%. The identities between the *Cyanobium* sp. and each of the two *P. ectocarpi* PKS gene sequences, also at the amino acid level, were of 45% and 47%, respectively. From NaPDoS phylogenetic analysis [15], the sequences from *P. ectocarpi* were found to bear homology and cluster with the KS domains from trans-AT type I PKSs VirF, CurF, JamJ and LnmJ (virginiamycin, curacin A, jamaicamides A and B, and leinamycin biosynthetic clusters, respectively). The sequence from *Cyanobium* sp. was shown to cluster with the starter KS domains from the biosynthetic gene clusters of curacin A (CurA) and jamaicamides A and B (JamE).

### Biological activity

While antiplasmodial activity was reported for **1** as a 1∶1 mixture with its two-carbon longer analogue [9], any information regarding its cytotoxicity towards human cells was not available. Therefore, we tested pure hierridin B to a panel of eight human cell lines (Table 1). When evaluated up to a maximum concentration of 30 µg mL^−1^ (82.3 µM), cytotoxicity was observed only to colon adenocarcinoma HT-29 cells (Table 1). A concentration-response assay (up to 100 µg mL^−1^) revealed its IC_50_ as 100.2 µM in this cell line (Fig. S2).

**Table 1:** Cytotoxicity of 1 towards a panel of human cell lines. (+ growth inhibition observed, − no growth inhibition observed).

Cell line	Type	Growth inhibition (IC_50_)
HepG2	hepatocellular carcinoma	− (n/a)
HT-29	colon adenocarcinoma	+ (100.2 µM)
MG63	osteosarcoma	− (n/a)
PNT2	normal prostate epithelium	− (n/a)
RKO	colon adenocarcinoma	− (n/a)
SHSY5Y	neuroblastoma	− (n/a)
SKBR3	breast adenocarcinoma	− (n/a)
T47D	breast ductal carcinoma	− (n/a)

## Discussion

### Biogenesis

**Figure 5:**
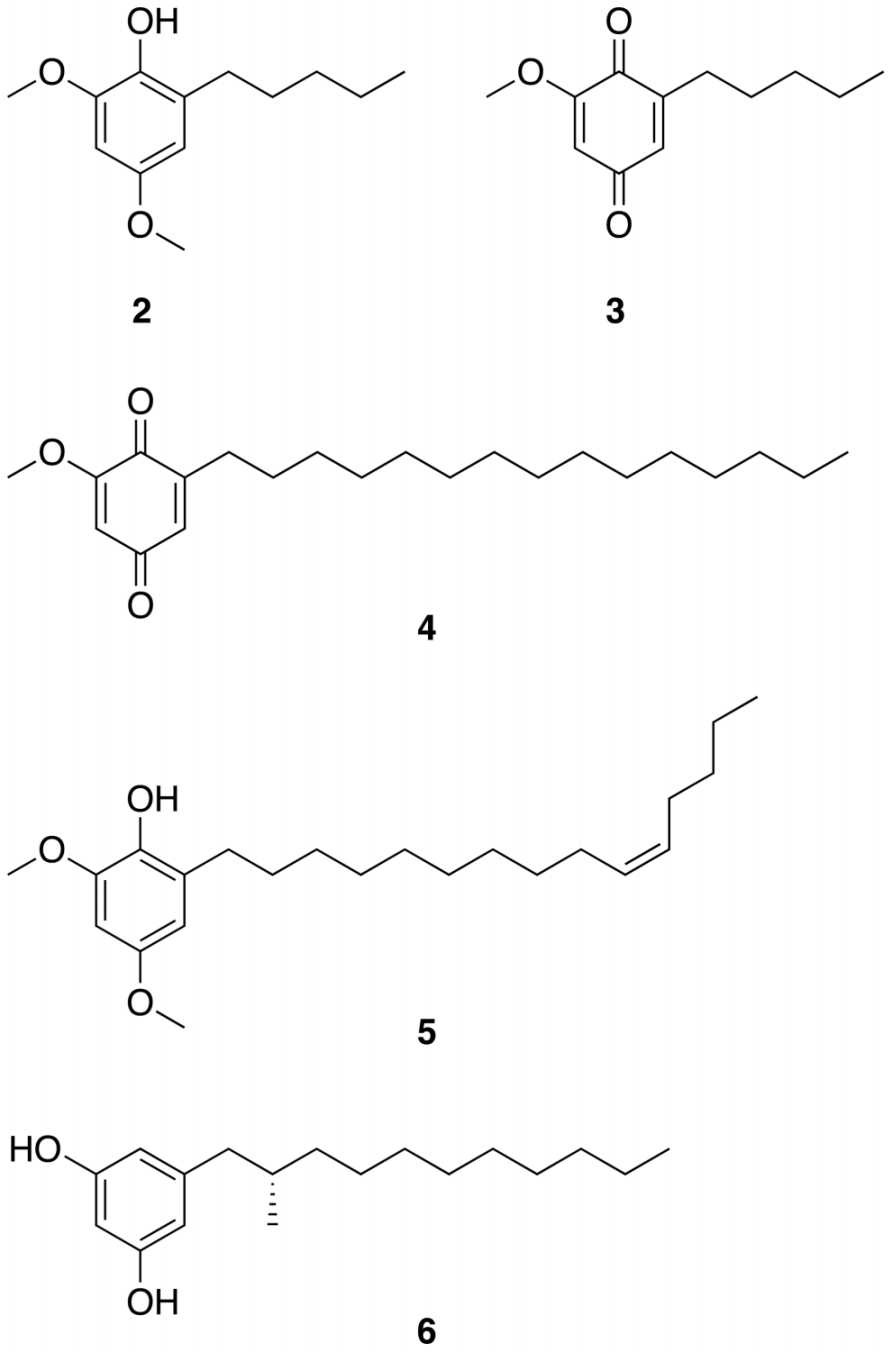
Selected natural products with structures related to that of compound 1. Miconidin methyl ether (**2**), primin (**3**), 2-methoxy-6-pentadecyl-1,4-benzoquinone (**4**), belamcandol A (**5**) and the resorcinol monomer of cylindrocyclophane F (**6**) are shown.

Feeding studies coupled with intermediate analysis by Horper and Marner [16], [17] on miconidin and some of its derivatives (including miconidin methyl ether **2** (Fig. 5), a shorter chain analogue of **1**) provided evidence for the polyketide nature of these metabolites, isolated from the plant *Primula obconica* [17]. The biosynthesis of **2** is thus proposed [16] to involve a triketoacid (2,4,6-trioxoundecanoic acid), which is cyclized, dehydrated and decarboxylated to yield 5-pentylbenzene-1,3-diol. This pathway intermediate leads to a variety of end products, including **2**, following methylation and oxidation. Another end-product of this putative pathway is the benzoquinone primin (**3**, Fig. 5), produced by the same plant [17]. Analogous long-chain quinones have been reported from the plant kingdom; for example, 2-methoxy-6-pentadecyl-1,4-benzoquinone (**4**, Fig. 5) from *Iris pseudacorus* seeds [18] features a pentadecyl chain, as seen in **1**. Belamcandol A (**5**, Fig. 5), found in the seeds of *Belamcanda chinensis* [19], is an unsaturated analogue of **1**. All of these long-chained metabolites generally can be explained by the biogenetic model put forward by Horper and Marner [16] for **2** and **3**. A similar biosynthetic origin is thus envisioned for **4**, **5** and **1**. In the present study, however, no evidence was found for the presence of any other end products or intermediates that could originate from an analogous biosynthetic route to **1**. Still, taking into account the small amount of compound **1** that we were able to purify, such metabolites may not have been detected during our isolation procedures. The alkyl side chain in **2** and **3** has been proposed [16] to originate from acetyl-CoA which is polymerized by a PKS assembly line, rather than hexanoic acid being used as a starter unit. The recently reported biosynthetic route to the cylindrocyclophanes in the filamentous heterocystous cyanobacterium *Cylindrospermum licheniforme* [20] involves an alkylresorcinol intermediate (**6**) that is structurally related to hierridin B. In this case, decanoic acid is used as the starter unit. The concerted action of two type I PKSs leads to a diketo intermediate, which is then cyclized by a type III PKS to **6** [20]. In the case of **1**, one can only speculate on whether palmitic acid primes this biosynthetic pathway, or whether a PKS is involved in the constructing the entire carbon framework. A polyketide origin for **1** is further supported by the fact that both hierridin B-producing cyanobacteria bear type I PKS machinery. Nevertheless, we were unable to find highly homologous KS domains shared by the two organisms, which would strengthen this hypothesis. It is unlikely that the PKS gene found in *Cyanobium* sp. LEGE 06113 is involved in the same biosynthetic transformation as any of the PKS genes found in the *P. ectocarpi* strain. However, it is possible that the three recovered KS domains correspond to different portions of the putative hierridin B pathway. The fact that all of these domains show highest homology to KS domains involved in curacin A and jamaicamides biosynthesis is consistent with such a scenario.

### Phylogenetic inferences

Our phylogenetic analysis, motivated by the aim of understanding how related the two hierridin B producer strains are to one another, revealed that *Cyanobium* sp. LEGE 06113 and *Phormidium ectocarpi* SAG 60.90 are clearly located in two distinct clades. Although these two clades emerged from the analysis as being more closely related to one another than with the other cyanobacterial lineages (Fig. 4), a common evolutionary lineage could not be established. Thus, it is improbable that the biosynthesis of this compound is deeply entrenched in the two lineages since their divergence. In fact, the findings of Shih et al. [4], point out that the genomes of some reference strains included in our phylogenetic study do not contain polyketide gene clusters, such as *Synechococcus* sp. PCC 7335 and the filamentous cyanobacterium *Prochlorothrix hollandica* PCC 9006 (axenic strain, co-identical with SAG 10.89 [21]). Both of these latter strains are placed between the two sub-clades that include the hierridin B producers. By contrast, *Synechococcus* sp. WH5701 and *Cyanobium* sp. 7001 (which belongs to the same clade as *Cyanobium* sp. LEGE 06113), and *Leptolyngbya* sp. PCC 7375 (closely related to *P. ectocarpi* SAG 60.90) do possess PKS clusters. Hence, as hypothesized for other secondary metabolites produced by cyanobacteria [22], [23], [24], horizontal transfer of the entire gene cluster may be responsible for the presence of the hierridin biosynthetic machinery in these two distinct phylotypes. It will thus be of considerable interest, in particular from the perspective of secondary metabolite evolution, to study the distribution of hierridin B among cyanobacteria. However, to achieve this it will be necessary to identify the cluster of genes involved in its biosynthesis.

### Biological activity

Papendorf et al. [9] isolated compound **1** guided by its antiplasmodial activity (low micromolar IC_50_). In the same study, the compound was tested but did not show antibacterial or anti-algal activity. It was also shown that **1** had no significant inhibitory activity in *in vitro* bioassays using HIV-1-reverse transcriptase or tyrosine kinase [9]. Antiplasmodial activity has been reported for the related metabolites **3** [25] and miconidin [26]. In this work, compound **1** showed selective activity, although of modest potency, towards the HT-29 cell line. The basis for this selectivity is, at the moment, unclear, but may indicate the existence of a unique target in this cell line and the possibility of a favorable therapeutic window. Still, the related benzoquinone irisquinone [18] did not reveal any such selectivity in the NCI-60 cancer cell line screening program (NSC Number 614642), in which it showed an average potency in the low-to-mid micromolar range. Other benzoquinones, such as **3** and its longer-chain analogue, 2-methoxy-6-heptyl-1,4-benzoquinone, have been associated with anticancer activity [27]. To our knowledge, no putative natural roles have been put forward for **1**. It has been found, however, that naturally occurring concentrations of miconidin and **3** are able to effectively deter feeding by insects [28]. Cyanobacteria serve as prey for several insect larvae, and members of the cyanobacterial genera *Fischerella* and *Hapalosiphon* have been reported to produce insecticidal metabolites [29]. However, the active components of these latter cyanobacteria bear no structural relatedness to **1**.

In conclusion, we report here the isolation and cytotoxicity of a bioactive secondary metabolite, hierridin B (**1**), from a marine picocyanobacterium, *Cyanobium* sp. LEGE 06113. Interestingly, this compound had been previously isolated from a phylogenetically distant filamentous cyanobacterium. Future efforts to elucidate the biosynthetic route to **1** will be of importance not only in the study of the evolution and distribution of this and other related natural products, but also to better understand the pathway cyanobacteria use to create these structures and to which end they are produced (i.e. from the eco-physiological point of view). Because *Cyanobium* spp. in general, and the strain used here in particular, exhibit high growth rates in laboratory cultures, and are thus being considered for biotechnological applications (e.g. [30], [31]), metabolite **1** can be considered as an easily accessible by-product from *Cyanobium* sp. LEGE 06113 biomass that may add to the value of its large scale culture.

## Materials and Methods

### Cyanobacterial strains

*Cyanobium* sp. LEGE 06113 was isolated from a sand sample in a *Sabellaria* sp. reef collected in the lower tide zone of an intertidal rocky shore (Aguda beach, Portugal, N 41° 02′ 58,35″, W 8° 39′ 19,22″). Field sampling at this location does not require, according to Portuguese law, any specific permission. In addition, the sampling did not involve any endangered or protected species. A monocyanobacterial culture of this strain is maintained in our culture collection (LEGE). For biomass production and subsequent chemical investigations, *Cyanobium* sp. LEGE 06113 cells were grown in 4 L cultures in Z8 medium [32] supplemented with 20 g L^−1^ NaCl, under a light/dark cycle of 14∶10 h and a photon irradiance of approximately 30 µmol m^−2^ s^−1^. Cells were harvested by centrifugation from nine of such cultures, rinsed with deionized water and freeze-dried. *Phormidium ectocarpi* SAG 60.90 was acquired from the Culture Collection of Algae at Goettingen University (SAG) and cultured in MN medium [33], under the light and temperature conditions described above. Microphotographs and morphometric measures of cultures of the two cyanobacterial strains were acquired in a BX41 light microscope equipped with a DP72 digital camera and Cell B image analysis software (Olympus, Tokyo, Japan).

### DNA extraction, amplification, sequencing and phylogenetic analysis

Biomass from exponential-phase, small-scale cultures of the two studied strains, *Cyanobium* sp. LEGE 06113 and *P. ectocarpi* SAG 60.90 was harvested (2 mL) by centrifugation. Total genomic DNA (gDNA) was extracted from the resulting biomass pellets using a commercial kit (PureLink™ Genomic DNA Mini Kit, Invitrogen, Carlsbad, CA, USA) and according to the manufacturer's instructions. A portion of the 16S rRNA gene was amplified by PCR with primers CYA 359F [34] and 1494Rc [35]. The primer pair 27F [35] and 781R [34] was used to amplify a different portion of the same gene in *P. ectocarpi* SAG 60.90, while we were unable to obtain this amplicon from *Cyanobium* sp. LEGE 06113. To amplify polyketide synthase (PKS) genes, primers DKF and DKR [14], which target the ketosynthase (KS) domain, were used. The amplified fragments were purified from the excised agarose gel slices (Cut&Spin columns, GRiSP, Porto, Portugal), cloned (pGEM-T® Easy vector, Promega, Madison, WI, USA), and transformed into OneShot® TOP10 cells (Invitrogen). Following purification (GenElute™ Plasmid Miniprep Kit, Sigma-Aldrich, St. Louis, MO, USA), plasmid DNA was sequenced (Macrogen, Inc., Seoul, Korea) using M13 primers. The resulting sequences were inspected for quality and deposited in GenBank (16S rRNA gene – accession numbers KC469577 and KC469578; PKS genes – accession numbers KF010866 to KF010868).

A 16S rRNA gene-based phylogenetic tree was constructed to infer the evolutionary relationship between the two hierridin B producing strains. Several available cyanobacterial sequences from unicellular and filamentous non-heterocystous “Bergey's reference strains” [11] were included in the analysis. Sequences from the best BLASTn hits in GenBank for *Phormidium ectocarpi* SAG 60.90 were also included in the multiple sequence alignment, which was performed in MEGA version 5 software package [36] using the ClustalW algorithm. The jModelTest 2.1.1 program [37] was used to evaluate which model of evolution best-fits our dataset. The corrected Akaike information criterion (AICc) was used to choose the optimal model of nucleotide substitution, which allowed us to select the GTR+Γ+I model (gamma shape parameter = 0.3860; number of gamma categories = 4; proportion of invariant sites = 0.3470). MrBayes version 3.1.2 [38] was then used for Bayesian inference (BI), using the same model and computing the parameter values indicated above. The phylogenetic tree reconstruction was performed using a random starting tree, while one cold and seven incrementally heated chains (temperature set 0.2) were run for 10^7^ generations, in two independent runs, with a tree sampling frequency of 100. The resulting consensus phylogeny was built from the last 75% of trees. MEGA 5 was also used to build a maximum-likelihood (ML) bootstrap tree (1000 replicates). The tree was reconstructed by using the same sequence alignment, model of evolution and parameters as the used for the Bayesian inferred tree. Gaps and ambiguously aligned positions were excluded (‘complete deletion’ option) from the analyses. The topologies retrieved from the two analyses were then evaluated using TreePuzzle 5.2 [39]. All the test comparisons indicated that the ML tree yielded the best topology for the dataset. The branch support values derived from the ML and BI analyses were then compared using TreeGraph 2 [40].

The PKS gene sequences obtained from both cyanobacterial strains were translated and submitted to the software tool NaPDoS (Natural Product Domain Seeker) [15], to identify the KS domains. The default parameters for KS domains were used in the analyses.

### Purification and dereplication of 1

Freeze-dried biomass from *Cyanobium* sp. LEGE 06113 (∼10 g, d.w.) was repeatedly extracted with a warm (<40°C) mixture of CH_2_Cl_2_∶MeOH (2∶1). The resulting crude extract (∼2.7 g) was subjected to normal-phase (silica gel 60, 0.015–0.040 mm, Merck KGaA, Damstadt, Germany) VLC, using a gradient from 100% hexane to 100% EtOAc to 100% MeOH. Nine fractions (A-I) were obtained and inspected by ^1^H NMR (500 MHz, Varian Inova). Fraction B (18.5 mg), putatively containing aromatic methoxy groups, was selected for further purification by semi-preparative RP-HPLC. The chromatographic system consisted of two 515 pumps and a 996 PDA detector (Waters, Milford, MA, USA), fitted with a Synergi Fusion-RP column (10 µm, 250×10 mm, Phenomenex, Torrance, CA, USA). A gradient program (flow of 3 mL min^−1^) was used for the separation, from 85% MeCN (aq) to 100% MeCN for 30 min, then 100% MeCN for 13 min, to afford metabolite **1** (0.6 mg, pale yellow amorphous solid, RT = 24.8 min). The ^1^H NMR (500 MHz, CDCl_3_) spectrum of **1** is available in Fig. S1.

Dereplication of known marine secondary metabolites was carried out with MarinLit (February 2011 update), by querying for the two methoxy groups and for a 1,2,3,5-tetrasubstituted aromatic moiety that was deduced from the *meta*-coupling (*J* = 2.8 Hz) of the two aromatic protons.

### GC-MS analysis

A TRACE GC Ultra (Thermo Fisher Scientific, Austin, TX, USA) gas chromatograph coupled with a Polaris Q ion trap mass spectrometer was used in the analysis. The system included an AS-3000 autosampler. A ZB-XLB capillary column (30 m×0.25 mm×0.25 µm) from Phenomenex® (Torrance, CA, USA) was selected for chromatographic separation. Ultra-pure grade Helium (Linde Sógas, Lisbon, Portugal; purity ≥99.999%) was used as a carrier gas (flow 1.0 mL min^−1^). The system was controlled by Xcalibur software, Version 1.3 (Thermo Fisher Scientific). Injections (2 µL) of **1** at a concentration of 2 mg L^−1^ were carried out in the splitless mode and injector temperature was 250°C. The compound was detected by mass spectrometry using full scan mode (*m/z* 80-500).

The column oven temperature was programmed as follows: initial temperature of 150°C and increased by 6°C min^−1^ to 295°C (held for 4 min). The mass spectrometer was operated in electron ionization (EI) mode at 70 eV with an external ionization source. A temperature of 285°C was used for the ion source and the electron multiplier was set at 1750 V (autotune to gain of 1×10^7^).

### Cell lines and cytotoxicity assays

All cell lines included in the study are of human origin. Hepatocellular carcinoma cell line HepG2, colon adenocarcinoma cell line HT-29, neuroblastoma cell line SH-SY5Y, breast carcinoma cell line T47D and the normal prostate cell line PNT2 were purchased from Sigma-Aldrich. SKBR3 breast adenocarcinoma and RKO colon carcinoma (ATCC) were a kind gift from Helena Fernandes (Faculdade de Medicina Dentária, University of Porto) and MG-63 osteosarcoma was obtained from the ATCC. Tumor cells were cultured in Dulbecco's modified Eagle medium (DMEM Glutamax) and the normal prostate cell line was cultured in minimal essential medium (α-MEM), supplemented with 10% fetal bovine serum (FBS), 2.5 µg mL^−1^ fungizone, penicillin-streptomycin (100 IU mL^−1^ and 100 µg mL^−1^, respectively). Cells were incubated in a humidified atmosphere with 5% of CO_2_, at 37°C.

The cellular viability was evaluated by the reduction of the 3-(4,5-dimethylthiazole-2-yl)-2,5-diphenyltetrazolium bromide (MTT) [41]. Cells were seeded in 96-well culture plates at a concentration of 10^4^ cells cm^−2^. After 24 h of adhesion, cells were exposed to 100 µL fresh medium supplemented with **1** to a final concentration of 3 and 30 µg mL^−1^, for a period of either 24, 48 and 72 hours. After incubation, cells were exposed to 10 µL of 0.5 mg/ml MTT. Following exposure, purple-colored formazan salts were dissolved in 100 µl DMSO and the absorbance measured at 550 nm in a microplate reader (Synergy HT, Biotek, USA). All tests were run in triplicate and averaged. A similar procedure was undertaken for determining the IC_50_ value of **1** in the cell line HT-29. In this case, however, cells were incubated for 48 h and the final concentrations of **1** in the culture medium ranged from 0.03 to 100 µg mL^−1^. Dose-response data was used to calculate the IC_50_ with the software GraphPad Prism v5.0 (GraphPad Software, La Jolla, CA, USA).

## Supporting Information

Figure S1 **^1^H NMR spectrum (500 MHz, CDCl_3_) of hierridin B (1).** Insert shows comparison with previously reported ^1^H NMR data (300 MHz, CDCl_3_) for the compound (Papendorf et al., 1998, *Phytochemistry* 49:2383–2386).
(TIF)

Figure S2 **Cytotoxicity dose-response curve of HT-29 cells exposed to hierridin B (1) (IC_50_ = 100.2 µM).**
(TIF)
