# Supplementary figures and images for: Antitumor Activity of Hierridin B, a Cyanobacterial Secondary Metabolite Found in both Filamentous and Unicellular Marine Strains
Source: PLoS One. 2013 Jul 29;8(7):e69562. doi: 10.1371/journal.pone.0069562 (PMC3726634; doi:10.1371/journal.pone.0069562)

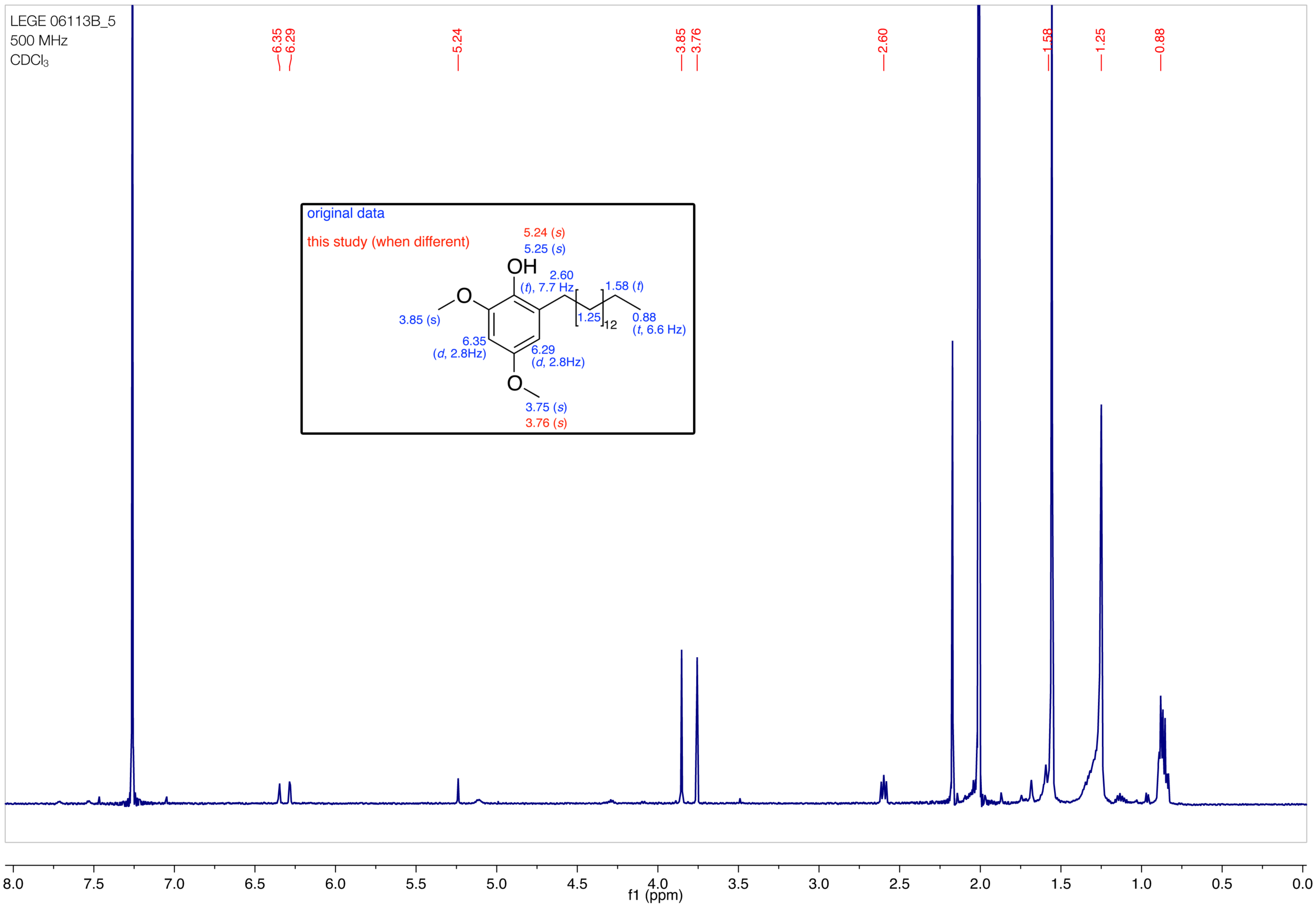

Supplement: Figure S1 — 1H NMR spectrum (500 MHz, CDCl3) of hierridin B (1). Insert shows comparison with previously reported 1H NMR data (300 MHz, CDCl3) for the compound (Papendorf et al., 1998, Phytochemistry 49:2383–2386). (TIF) [file pone.0069562.s001.tif]

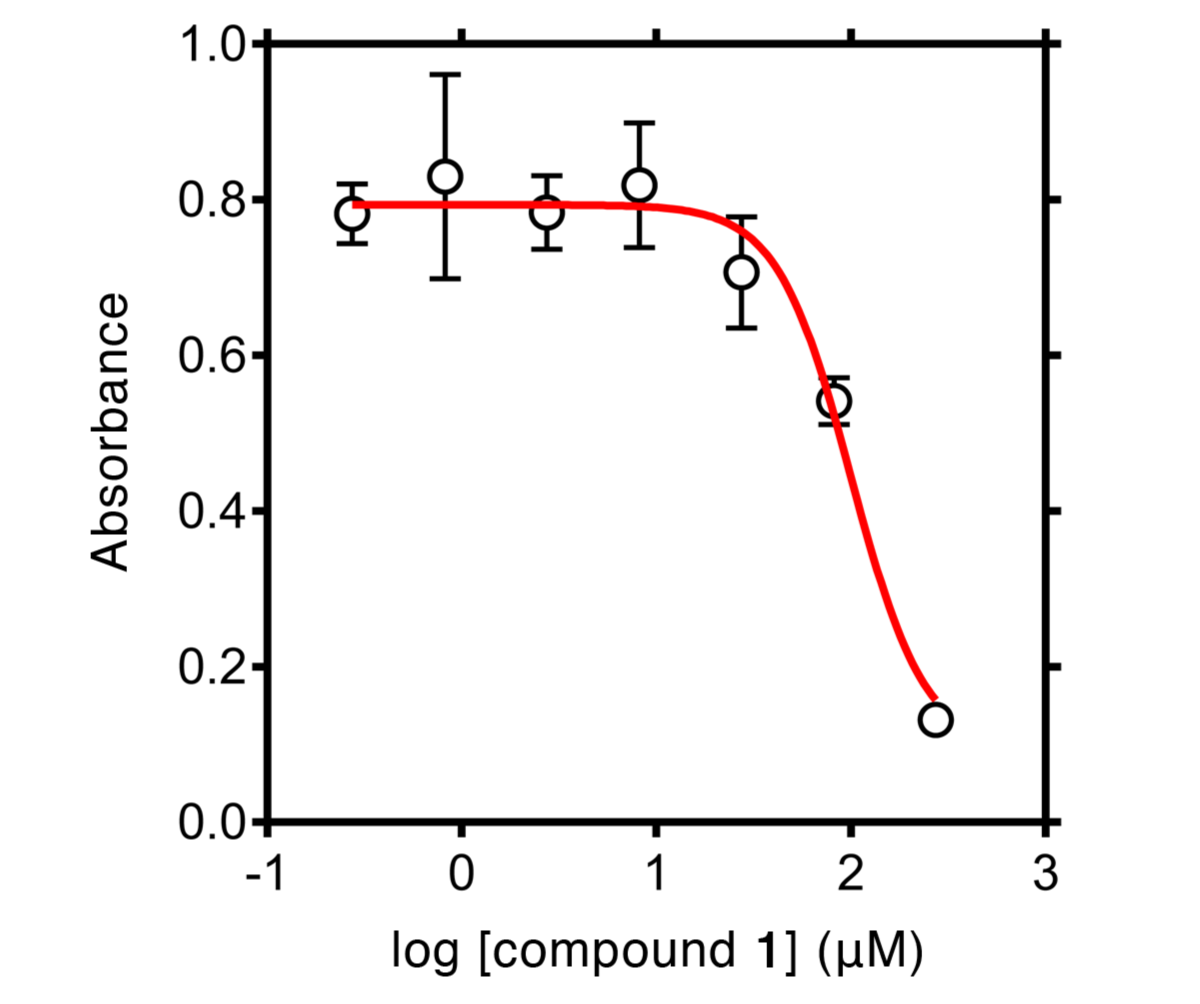

Supplement: Figure S2 — Cytotoxicity dose-response curve of HT-29 cells exposed to hierridin B (1) (IC50 = 100.2 µM). (TIF) [file pone.0069562.s002.tif]
